# Comprehensive Theoretical Studies on the Reaction of 1-Bromo-3,3,3-trifluoropropene with OH Free Radicals

**DOI:** 10.3390/molecules18077873

**Published:** 2013-07-04

**Authors:** Meiling Zhang, Ce Song, Yan Tian

**Affiliations:** 1 School of Biomedical Engineering, Tianjin Medical University, Tianjin 300070, China; 2 College of Basic Medical Sciences, Tianjin Medical University, Tianjin 300070, China; 3 Hefei National Laboratory of Physical Sciences at the Microscale, University of Science and Technology of China, Hefei 230026, Anhui, China; E-Mail: csong@ustc.edu.cn; 4 Department of Theoretical Chemistry and Biology, School of Biotechnology, Royal Institute of Technology, Stockholm SE-10691, Sweden; 5 School of Science, Anhui Agricultural University, Hefei 230036, Anhui, China; E-Mail: tianyan@ahau.edu.cn

**Keywords:** quantum chemical calculations, reaction mechanism, transition states, potential energy surface, reaction rate constants

## Abstract

The potential energy surfaces (PES) for the reaction of 1-bromo-3,3,3-trifluoropropene (CF_3_CHCBrH) with hydroxyl (OH) free radicals is probed theoretically at the CCSD/aug-cc-pVDZ//B3LYP/6-311++G(d,p) level of theory. All the possible stationary and first-order saddle points along the reaction paths were verified by the vibrational analysis. The calculations account for all the product channels. Based on the calculated CCSD/aug-cc-pVDZ potential energy surface, the possible reaction mechanism is discussed. Six distinct reaction pathways of 1-bromo-3,3,3-trifluoropropene (BTP) with OH are investigated. The geometries, reaction enthalpies and energy barriers are determined. Canonical transition-state theory with Wigner tunneling correction was used to predict the rate constants for the temperature range of 290–3,000 K without any artificial adjustment, and the computed rate constants for elementary channels can be accurately fitted with three-parameter Arrhenius expressions. OH addition reaction channel and the H atom abstraction channels related to the carbon-carbon double bond are found to be the main reaction channels for the reaction of 1-bromo-3,3,3-trifluoropropene (CF_3_CHCBrH) with hydroxyl (OH) free radicals while the products leading to CF_3_CHCH + BrOH and COHF_2_CHCBrH + F play a negligible role.

## 1. Introduction

Ever since Halon 1301 (CF_3_Br) was viewed as a serious ozone-depleting substance, the development and manufacture of non-ozone-depleting replacement agents has attracted wide research interests. As Halon alternatives, inert gases and halocarbon fire extinguishing agent are now commonly used because of their cleanliness, effectiveness and non-conductive characteristics. One class of suppressants currently under consideration is the bromofluoroalkene family [1]. The presence of a carbon-carbon double bond is expected to render these substances highly reactive toward the hydroxyl radical, OH, resulting in an extremely short tropospheric lifetime, thereby limiting their delivery of bromine to the stratosphere where it can participate in ozone-destroying catalytic reactions. At the same time, the presence of a Br atom suggests that these compounds will also have a high degree of fire suppression efficiency [2], so the bromocarbon-containing carbon-carbon double bond is tropodegradable and maintains high fire suppression efficiency [3].

1-Bromo-3,3,3-trifluoropropene (BTP) is composed with molecular structures containing both a carbon-carbon double bond and a bromine atom. Some researchers have proved that the ozone destroying potency and the globe potency of BTP is zero [4], so BTP represents one of the greatest hopes for the eventual identification of a practical Halon 1301 replacement. There have been several recent studies that have attempted to deduce the chemical behavior of BTP under different conditions [5,6,7,8,9,10,11,12]. BTP suppresses flames by both chemical and physical mechanisms. BTP is able to extinguish flames physically by removing thermal energy from the flame. It is thought that BTP suppresses flames chemically by removing important species that are necessary for flame propagation. The reactions of OH are extraordinarily important in atmospheric chemistry and in combustion science. However, the reaction mechanism between BTP and hydroxyl (OH) free radical is still unclear except some studies on the 1-bromo-3,3,3-trifluoropropene (BTP) thermal decomposition [11] and experimental study of the fire-extinguishing effectiveness of 1-bromo-3,3,3-trifluoropropene [12].

In this work, the CCSD/aug-cc-pVDZ//B3LYP/6-311++G(d,p) theory was used for the first time to directly compute the reaction barriers of all reaction channels without any further adjustments of the energies. The energetics of these reactions was used together with the transition state theory (TST) [13,14,15,16,17,18,19,20] to compute rate constants in the temperature range 290–3,000 K without the need of empirical and complicated extrapolation procedures based on the low temperature data. Possible reaction channels between BTP and OH are considered and discussed exhaustively. The relative importance of the elementary pathways is discussed quantitatively. The computed results provide the best available estimate for BTP extinguishing mechanism in use of research in the related field, and the comparison with future experimental measurements can provide a test to the predictive ability of the reaction computational theory.

## 2. Results and Discussion

**Figure 1:**
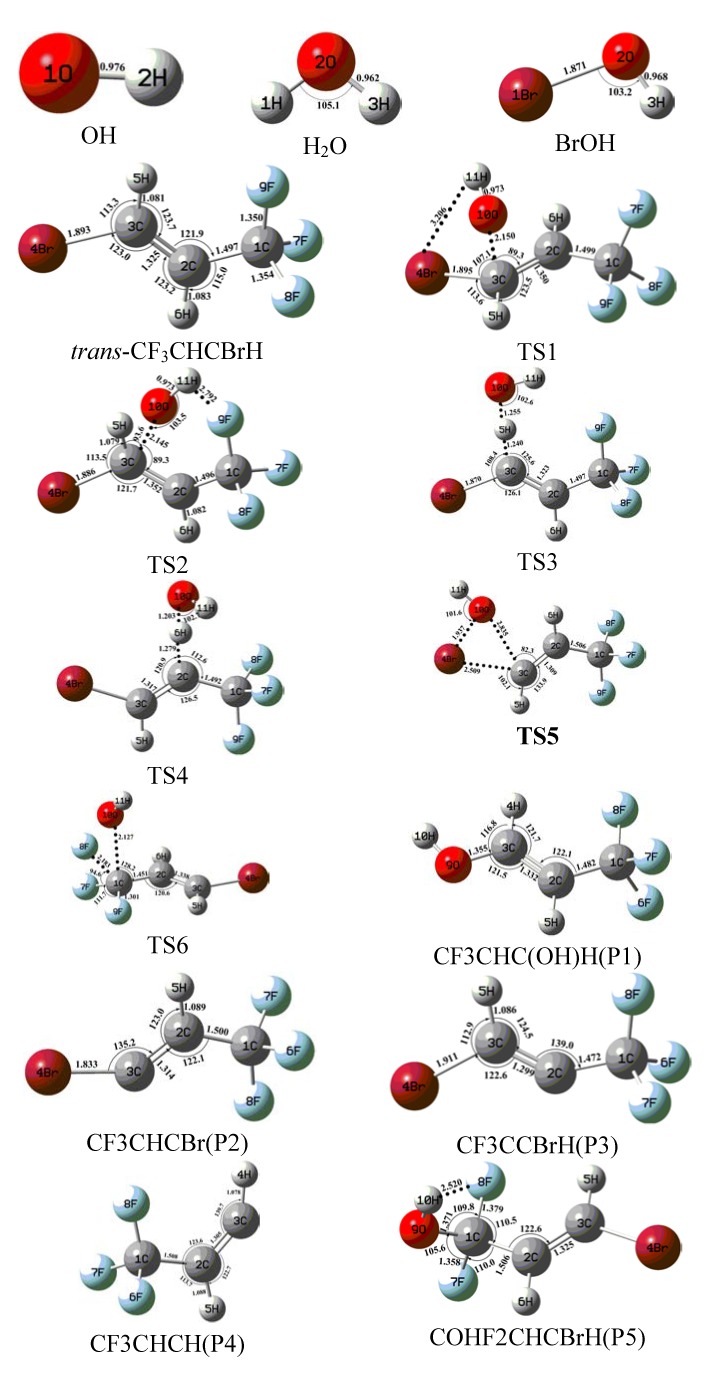
Optimized structures of various species involved in the reaction of trans-CF_3_CHCBrH with OH free radical at the B3LYP/6-311++G(d,p) level. Bond distances are in angstroms and angles are in degree.

Shown in Figure 1 are the geometric structures for the reactants, transition states, and products involved in the reactions as optimized at the B3LYP/6-311++G(d,p) level. For all the species involved, the results of the B3LYP/6-311++G(d,p) and CCSD/aug-cc-pVDZ//B3LYP/6-311++G(d,p) energies, the zero-point vibrational energies obtained at the B3LYP/6-311+ +G** level, and the enthalpies at the CCSD/aug-cc-pVDZ//B3LYP/6-311++G(d,p) level can be found in Table 1. 

**Table 1:** The relative energies (in kcal/mol), ΔE (DFT), ΔE (CCSD) and ΔH_298_(CCSD), as well as the zero-point vibration energies (ZPVEs) of species involved in the BTP reaction with OH.

Column heading	Species	ΔE(CCSD)	ΔE(DFT)	ZPE	ΔH_298_(CCSD)
Reactants	*trans*-CF_3_CHCBrH + OH	0.0	0.0	34.9	0.0
Transition states	TS1	1.5	−1.4	36.5	2.3
	TS2	2.1	−1.0	36.4	2.8
	TS3	11.1	3.5	32.8	8.4
	TS4	13.9	6.7	32.5	11.1
	TS5	42.3	36.5	36.5	42.6
	TS6	78.6	59.8	36.3	79.2
Products	CF_3_CHC(OH)H +Br (P1)	−38.5	−38.8	38.2	−35.3
	CF_3_CHCBr+H_2_O (P2)	−5.9	−8.3	34.9	−5.6
	CF_3_CCBrH+H_2_O (P3)	0.0	−3.3	34.5	0.1
	CF_3_CHCH+BrOH (P4)	33.2	31.2	34.9	32.8
	COHF_2_CHCBrH+F (P5)	13.9	16.0	37.1	15.9

**Figure 2:**
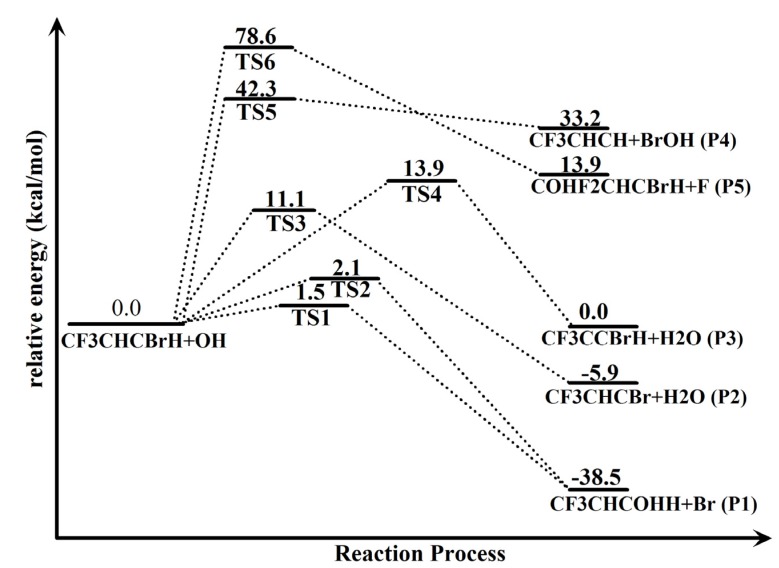
Potential energy surface for the reaction of trans-CF_3_CHCBrH with OH free radical based on the relative energies obtained at the CCSD/aug-cc-pVDZ//B3LYP/6-311++G(d,p) level of theory. The energies of reactants CF_3_CHCBrH + OH are set to zero.

The stationary geometries were confirmed by harmonic vibration frequency analysis, *i.e.*, the reactants and products all possess real frequencies, whereas the transition structures possess only one imaginary frequency. The energy profiles of the potential energy surface for the reactions of 1-bromo-3,3,3-trifluoropropene (CF_3_CHCBrH) with OH are plotted in Figure 2.

**Figure 3:**
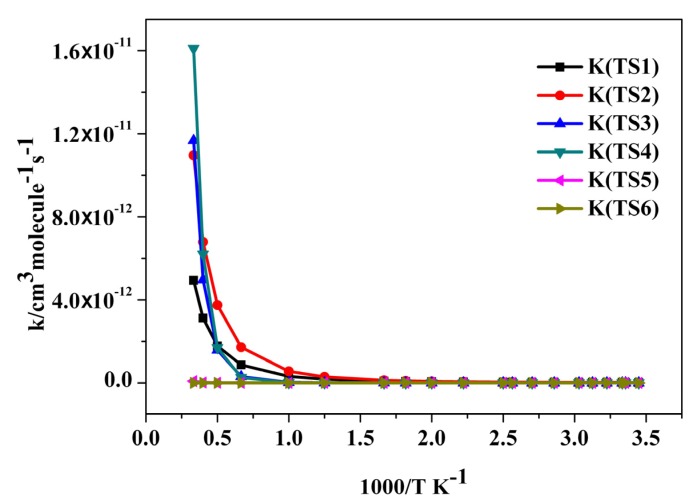
Theoretical rate constants for the reactions of trans-CF_3_CHCBrH with OH free radical over the temperature range of 290–3,000 K.

The calculated rate constants of the main reaction channels for the trans-CF_3_CHCBrH + OH reaction by the TST theory are showed in Figure 3. Roughly six distinct reaction pathways (A–F) for the reaction of BTP with OH (see Figure 2) are investigated in the following and, unless noted otherwise, the CCSD/aug-cc-pVDZ//B3LYP/6-311++G(d,p) energies are used.

### 2.1. Conformational Surface

Figure 1 is the calculated equilibrium structure of BTP obtained from the B3LYP/6-311++G(d,p) optimization. The backbone of the molecule is formed by a chain of three carbon atoms. 1-Bromo-3,3,3-trifluoropropene (BTP) is composed with molecular structures containing both a carbon-carbon double bond and a bromine atom. In addition, two orientations of the hydrogen group in the BTP (CF_3_CHCBrH) are involved, *i.e.*, the *cis*-CF_3_CHCBrH and *trans*-CF_3_CHCBrH, the detailed structure information please reference our previous work in ref. [11]. When the two hydrogen groups are oriented in the same direction relative to the double bond, the diastereomer is referred to as *cis*-CF_3_CHCBrH, whereas, when the hydrogen groups are oriented in opposing directions relative to the double bond, the diastereomer is referred to as *trans*-CF_3_CHCBrH. The energy difference between *cis*- and *trans*-CF_3_CHCBrH is determined to be 3.0 kcal/mol, with *trans*-CF_3_CHCBrH being slightly favored. The zero-point vibrational energies as well as the thermal correction energies by the B3LYP/6-311++G(d,p) calculations were found to be essentially the same for *cis*- and *trans*-CF_3_CHCBrH. The vibrational frequencies for the reactant, transition state and products involved in the reaction of 1-bromo-3,3,3-trifluoropropene (CF_3_CHCBrH) with OH are available by contacting the authors.

### 2.2. Reaction Mechanism of Trans-CF_3_CHCBrH + OH

When OH radical and trans-CF_3_CHCBrH approach each other, various possible products may be formed when OH radical interacts with different atoms of the CF_3_CHCBrH molecules in the reaction. These different products are CF_3_CHC(OH)H + Br (P1), CF_3_CHCBr + H_2_O (P2), CF_3_CCBrH + H_2_O (P3), CF_3_CHCH + BrOH (P4) and COHF_2_CHCBrH + F (P5). The mechanisms of their formation are as follows:

Pathway A

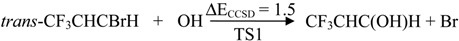
(1)

The O atom of OH attacks the C3 atom of the unsaturated trans-CF_3_CHCBrH firstly and at the same time the Br atom on the trans-CF_3_CHCBrH departs to form the products CF_3_CHC(OH)H + Br. In this process, two possible reaction channels are detected because of the different attacking direction of the hydroxyl. In pathway A, the OH first addition to the carbon atom C3 related to the unsaturated double bond with the H atom on the OH directing to the Br atom of the trans-CF_3_CHCBrH via TS1 (see Figure 1 and Figure 2) with an imaginary frequency of 232i, whose barrier height (see Figure 2) is only 1.5 kcal/mol. Then the bromine departs from the C3 atom forming the products CF_3_CHC(OH)H + Br. The energies of the products CF_3_CHC(OH)H + Br are 38.5 kcal/mol below than that of the reactants and this reaction channel is the most exothermic pathway.

Pathway B

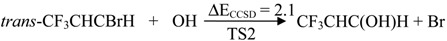
(2)

Analogous to pathway A, the products CF_3_CHC(OH)H + Br may be produced in another way. The O atom of OH attacking the C3 atom of the unsaturated *trans*-CF_3_CHCBrH in a different direction with the H atom on the OH directing to the F9 atom instead of directing to the Br atom of the *trans*-CF_3_CHCBrH via TS2 (see Figure 1) with a slightly higher energy barrier of 2.1 kcal/mol than the pathway A. TS2 is a HO-BTP adduct with an imaginary frequency of 264i, whose barrier height (see Figure 2) is only 0.6 kcal/mol higher than TS1. Due to low reaction barriers and the most exothermic reaction, the two channels mentioned above play important roles in the overall reaction of *trans*-CF_3_CHCBrH with OH.

Pathway C

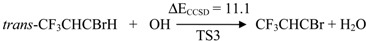
(3)

The O atom of OH may abstract the H atom of the trans-CF_3_CHCBrH molecule to form CF_3_CHCBr + H_2_O via TS3 directly, whose barrier height (see Figure 2) is 11.1 kcal/mol. As shown in Figure 1, the breaking bond C3–H5 in TS3 is stretched to 1.240 Å, which is about 0.159 Å longer than that in parent trans-CF_3_CHCBrH molecule. The forming O10–H5 bond is 1.255 Å, which is 0.293 Å longer than that in the H_2_O product. The transition state TS3 has simple structure of C1 symmetry and is a true first-order saddle point with an imaginary frequency of 1325i. From Table 1 and Figure 2, the H-atom abstraction reaction of *trans*-CF_3_CHCBrH by OH is an exothermic reaction. The total energy of products CF_3_CHCBr + H_2_O relative to the reactants *trans*-CF_3_CHCBrH + OH is −5.9 kcal/mol.

Pathway D

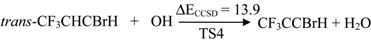
(4)

The free radical OH not only can abstract the H atom on C3 of the carbon-carbon double bond in the *trans*-CF_3_CHCBrH molecule to form CF_3_CHCBr + H_2_O, but can also attract the H atom on C2 of the carbon-carbon double in the *trans*-CF_3_CHCBrH molecule to form the products CF_3_CCBrH + H_2_O via TS4 with a slightly higher barrier height of 13.9 kcal/mol, which is 2.8 kcal/mol higher than the H abstract channel on C3. The transition state TS4 has a simple structure of C1 symmetry and is a true first-order saddle point with an imaginary frequency of 1487i. The breaking C2–H6 bond in TS4 is 1.279 Å longer than that of *trans*-CF_3_CHCBrH. Seen from Figure 1, the forming O10–H6 bond is 1.203 Å, which is 0.241 Å longer than that in the H_2_O product. The energy of the products CF_3_CCBrH + H_2_O is just same with that of the reactants in this reaction channel.

Pathway E

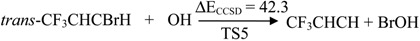
(5)

The direct Br atom abstraction by OH free radical from trans-CF_3_CHCBrH will produce the products CF_3_CHCH + BrOH via a C3–Br4 bond fission transition state TS5. TS5 has a relatively high barrier height of 42.3 kcal/mol, which is 31.2 kcal/mol higher than the direct H abstract channel on C3. The C3–Br4 bond length is elongated by 0.616 Å in TS5 while the C2–C3 bond is shortened by 0.016 Å. TS5 has an imaginary frequency of 311i. The energy of CF_3_CHCH + BrOH is 33.2 kcal/mol higher than that of the reactants *trans*-CF_3_CHCBrH + OH. In view of the energetic, the energy barrier height of the Br abstract channel lies above that of the H abstract channel by 28.4 kcal/mol and 31.2 kcal/mol on C2 and on C3 respectively, the former is kinetically unimportant.

Pathway F

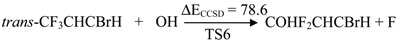
(6)

When the O atom of OH attacks the C atom of *trans*-CF_3_CHCBrH, other products COHF_2_CHCBrH + F may be produced via the transition state TS6. TS6 exhibits a simple structure of C1 symmetry and is a true first-order saddle point with an imaginary frequency of 398i. O atom of OH attacks the C atom of the *trans*-CF_3_CHCBrH, kicking off one F atom on the *trans*-CF_3_CHCBrH. As can be seen from Figure 1, the bond length of C1–F8 in TS6 is stretched to be 2.183 Å, *i.e.*, it is a loose bond. The forming bond C1–O10 is 2.127 Å, which is 0.756 Å longer than that in the COHF_2_CHCBrH product. The formation of COHF_2_CHCBrH and F is endothermic by 13.9 kcal/mol. However, the energy barrier for the reaction is rather high, 78.6 kcal/mol, which is the highest energy barrier reaction in all the channels of the reaction *trans*-CF_3_CHCBrH with OH. Clearly, this addition-elimination channel should play a negligible role in the reaction.

### 2.3. Reaction Rate Results

Due to the large differences in the reaction barrier heights of different reaction channels, the rate constant for the *trans*-CF_3_CHCBrH + OH reaction can be viewed as contributed mainly from the HO-BTP adduct channel and H atom abstraction channels related to the unsaturated carbon-carbon double bond, *i.e.* lead to the products CF_3_CHC(OH)H +Br, CF_3_CHCBr+H_2_O and CF_3_CCBrH+H_2_O for temperatures up to 1,000 K.

Figure 3 shows the calculated rate constants for the trans-CF_3_CHCBrH + OH reaction. The calculated rate constants exhibit a typical non-Arrhenius behavior. This non-Arrhenius behavior has frequently been observed in radical-molecule reactions studied over wide temperature ranges [21]. The calculated rate constants are fitted to a three-parameter formula over the temperature range of 290–3,000 K and are given in units of cm^3^ molecule^−1^ s^−1^ as the following expressions:

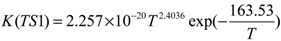
(7)

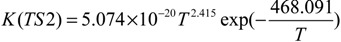
(8)

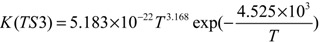
(9)

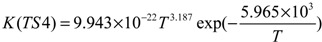
(10)

The products of TS5 and TS6 channels have a total energy higher than the energy of the *trans*-CF_3_CHCBrH + OH by a minimum of 13.9 kcal/mol and the reaction barrier is at least 42.3 kcal/mol by the two means. This is higher than the barrier height of 1.5 kcal/mol of the TS1 reaction channel by almost 40.8 kcal/mol and is well beyond the theoretical uncertainty. Therefore, our calculations have conclusively demonstrated that the TS5 results to CF_3_CHCH + BrOH channel and TS6 producing the COHF_2_CHCBrH + F channel of the reaction *trans*-CF_3_CHCBrH + OH is negligible in comparison with the first four reaction channels lead to the products CF_3_CHC(OH)H + Br, CF_3_CHCBr+H_2_O and CF_3_CCBrH + H_2_O temperature up to 1,000 K within a temperature range of our study. It is expected that the accurate future experiment measurement is very helpful in validating the theoretical model.

## 3. Computational Methods

The geometries of all reactants, products and transition state structures have been fully optimized using the B3LYP [22,23] method with the 6-311++G(d,p) [24] basis set. Tran *et al.* have previously reported the success of the B3LYP method in predicting geometries of unsaturated chain structures, and this method produces optimized structures, at low computational cost, that compared favorably with higher level calculations [25]. Harmonic vibrational frequencies have been calculated at the same level to determine the nature of the various stationary points as well as the zero-point vibrational energies (ZPVEs). The stationary geometries were confirmed by harmonic vibration frequency analysis, *i.e.*, the reactants and products possess all real frequencies, whereas the transition structures possess only one imaginary frequency. Connections between reactants, transition structures and products were confirmed by intrinsic reaction coordinate (IRC) [26,27,28,29] calculations at the B3LYP/6-311++G(d,p) level. To improve the accuracy of the energies, subsequent single-point calculations were performed at the coupled-cluster level of theory with single and double excitations (CCSD) [30] with a diffuse functions basis set aug-cc-pVDZ [31].

The CCSD/aug-cc-pVDZ//B3LYP/6-311++G(d,p) theory was used for the reaction rate calculations. In order to find which reaction channel dominates kinetically at different temperatures in the reactions, the canonical transition state theory [13,14,15,16,17,18,19,20] (TST) including semiclassical multiplicative tunneling correction factors was used to predict the temperature dependence of the rate constants. Accordingly, the rate constants, k(T), were computed using the following expression:

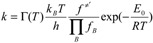
(11)
where Г*(T)* is the transmission coefficient to correct the tunneling effect at temperature T, E_0_ is the classical energy barrier including ZPE, *i.e.*, the enthalpy difference between reactants and transition states at 0 K, 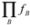 are the continued product of the reactions partition function, ƒ^≠^^′^ are the partition function of the transition state, k_b_ is the Boltzman’s constant, and h is the Planck’s constant.

The transmission coefficient was calculated by the Wigner method [32] as:

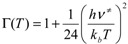
(12)
where ν^≠^ is the imaginary frequency at the saddle point. Choosing the simple and inexpensive Wigner method instead of the more sophisticated and computationally demanding algorithms such as the ones developed by Truhlar [33,34,35,36,37] and Miller [38,39] seems to be appropriate here as the tunneling corrections applied to the rate constants at typical combustion temperatures (typically above 2,000 K) are small to moderate (≤2) [40]. All the CCSD calculations were performed with the MOLPRO package [41] and all the B3LYP calculations were carried out with Gaussian 09 program [42].

## 4. Conclusions

Our CCSD/aug-cc-pVDZ//B3LYP/6-311++G(d,p) calculations provided detailed potential energy profile for the 1-bromo-3,3,3-trifluoropropene (CF_3_CHCBrH) with hydroxyl (OH) free radical reaction. Six distinct reaction pathways of 1-bromo-3,3,3-trifluoropropene (BTP) with OH are investigated. The reaction enthalpies and energy barriers are determined. From the energetics, the most feasible reaction pathways for 1-bromo-3,3,3-trifluoropropene with OH are those that related to the unsaturated carbon-carbon double bond, *i.e.* lead to the products CF_3_CHC(OH)H + Br, CF_3_CHCBr + H_2_O and CF_3_CCBrH + H_2_O. Considering the extreme high flame temperature, the first four reaction pathways are competitive. 

The rate constants for the main reaction channels were calculated by the TST theory. For the temperature range of 290–3,000 K, the calculated rate constants exhibit a typical non-Arrhenius behavior. Future experiment capable of reliably assessing the relative importance of the elementary reactions of the model combustion processes is necessary.

## References

[1] Proceedings of Halon Options Technical Working Conference.

[2] Saso Y., Ogawa Y., Saito N. (1999). Binary CF3Br- and CHF3-inert flame suppressants: effect of temperature on the flame inhibition effectiveness of CF3Br and CHF3. Combust Flame..

[3] Mather J.D., Robert E. Troprodegradable bromocarbon extinguishants-compound selection and testing issues. http://fire.nist.gov/bfrlpubs/fire02/PDF/f02120.pdf.

[4] Lifke J., Martinez A., Tapscott R.E., Mather J.D. Tropodegradable bromocarbon extinguishants. http://www.bfrl.nist.gov/866/NGP/publications/Tropo_Final_Rpt.pdf.

[5] Mather J.D., Tapscott R.E. Tropodegradable bromocarbon extinguishants—progress overview. Proceedings of the Halon Options Technical Working Conference.

[6] Gann R.G. Next-generation Fire Suppression Technology Program (NGP): Technical Highlights. Proceedings of the Halon Options Technical Working Conference.

[7] Tapscott R.E., Mather J.D., Moore T.A. (1998). Clean, Tropodegradable Agents with Low Ozone Depletion and Global Warming Potentials to Protect Against Fires and Explosions. U.S. Patent.

[8] Nyden M.R., Yang J.C., Mather J.D. Screening of candidate fire suppressants. Proceedings of the Halon Options Technical Working Conference.

[9] Tapscott R.E., Heinonen E.W., Lac J.L., Mather J.D., Moore T.A. Tropodegradable Bromocarbons as Halon Replacements, Proceedings of Halon Options Technical Working Conference.

[10] Grzyll L.R., Back D.D. (1997). Development of Quantitative Structure-property Relationships for Tropodegradable Halocarbon Fire Suppression Agents, Final Report, SSG Subtask 3.20, Subcontract S-5000.48, ARA, Inc., Tyndall Air Force Base, Florida, Mainstream Engineering Corporation, Rockledge. Florida. Mar..

[11] Zhang M.L., Lin Z.J. (2009). Ab initio studies of the thermal decomposition pathways of 1-bromo-3,3,3-trifluoropropene. J. Mol. Struc.-Theochem..

[12] Zhang Y.F., Jin X., Liao G.X. (2007). Experimental study of the fire-extinguishing effectiveness of 1-bromo-3,3,3-trifluoropropene/nitrogen mixtures. J. Fire Sci..

[13] Johnston H.S. (1966). Gas-Phase Reaction Rate Theory.

[14] Laidler K.J. (1969). Theories of Chemical Reaction Rates.

[15] Weston R.E., Schwartz H.A. (1972). Chemical Kinetics.

[16] Rapp D. (1972). Statistical Mechanics.

[17] Nikitin E.E. (1974). Theory of Elementary Atomic and Molecular Processes in Gases.

[18] Smith I.W.M. (1980). Kinetics and Dynamics of Elementary Gas Reactions.

[19] Steinfeld J.I., Francisco J.S., Hase W.L. (1989). Chemical Kinetics and Dynamics.

[20] Louis F., Gonzalez C.A., Sawerysyn J.P. (2003). Ab Initio Study of the Oxidation Reaction of CO by ClO Radicals. J. Phys. Chem. A.

[21] Garrett B.C., Truhlar D.G., Bowman J.M., Wagner A.F., Robie D., Arepalli S., Presser N., Gordon R.J. (1986). Ab initio predictions and experimental confirmation of large tunneling contributions to rate constants and kinetic isotope effects for hydrogen atom transfer reactions. J. Am. Chem. Soc..

[22] Becke A.D. (1992). Density-functional thermochemistry. II. The effect of the Perdew-Wang generalized-gradient correlation correction. J. Chem. Phys..

[23] Lee C., Yang W., Parr R.G. (1988). Development of the Colle-Salvetti correlation-energy formula into a functional of the electron density. Phys. Rev. B.

[24] Frisch M.J., Pople J.A., Binkley J.S. (1984). Self-consistent molecular orbital methods 25. Supplementary functions for Gaussian basis sets. J. Chem. Phys..

[25] Tran K.M., McAnoy A.M., Bowie J.H. (2004). Do the interstellar molecules CCCO and CCCS rearrange when energised?. Org. Biomol. Chem..

[26] Fukui K. (1981). The path of chemical reactions - the IRC approach. Acc. Chem. Res..

[27] Page M., Mclver J.W. (1988). On evaluating the reaction path Hamiltonian. J. Chem. Phys..

[28] Gonzalez C., Schlegel H.B. (1989). An improved algorithm for reaction path following. J. Chem. Phys..

[29] Gonzalez C., Schlegel H.B. (1990). Reaction Path Following in Mass-Weighted Internal Coordinates. J. Phys. Chem..

[30] Purvis G.D., Bartlett R.J. (1982). A full coupled-cluster singles and doubles model: The inclusion of disconnected triples. J. Chem. Phys..

[31] Kendall R.A., Dunning T.H., Harrison R.J. (1992). Electron affinities of the first-row atoms revisited. Systematic basis sets and wave functions. J. Chem. Phys..

[32] Wigner E.P. (1932). Über das Überschreiten von Potentialschwellen bei chemischen Reaktionen. Z. Phys. Chem. B.

[33] Garret B.C., Truhlar D.G. (1979). Semiclassical tunneling calculations. J. Phys. Chem..

[34] Garret B.C., Truhlar D.G. (1984). WKB approximation for the reaction-path Hamiltonian: Application to variational transition state theory, vibrationally adiabatic excited-state barrier heights, and resonance calculation. J. Chem. Phys..

[35] Skodke R.T., Garret B.C., Truhlar D.G. (1981). A General Small-Curvature Approximation for Transition-State-Theory Transmission Coefficients. J. Phys. Chem..

[36] Skodje R.T., Garret B.C., Truhlar D.G. (1982). Vibrationally Adiabatic Models for Reactive Tunneling. J. Chem. Phys..

[37] Garret B.C., Truhlar D.G., Grev R.S., Magnuson A.W. (1980). Improved Treatment of Threshold Contributions in Variational Transition State Theory. J. Chem. Phys..

[38] Miller W.H., Shi S.H. (1981). Unified semiclassical perturbation and infinite order sudden approximation, with application to the reaction path hamiltonian model. J. Chem. Phys..

[39] Miller W.H., Smith F.T. (1978). Semiclassical Perturbation Theory of Electron-Molecule Collisions. Phys. Rev. A.

[40] Bell R.P. (1980). The Tunnel Effect in Chemistry.

[41] Werner H.J., Knowles P.J., Amos R.D., Bernhardsson A., Berning A., Celani P., Cooper D.L., Deegan M.J.O., Dobbyn A.J., Eckert F. (2002). MOLPRO package, A package of ab initio programs.

[42] Frisch M.J., Trucks G.W., Schlegel H.B., Scuseria G.E., Robb M.A., Cheeseman J.R., Scalmani G., Barone V., Mennucci B., Petersson G.A. (2010). Gaussian 09, revision B.01.

